# Long-term results of arthroscopic repair of type II SLAP lesions in sports: assessment of return to pre-injury playing level and critical risk factors for complication

**DOI:** 10.1007/s00590-023-03677-w

**Published:** 2023-08-13

**Authors:** G. Della Rotonda, A. Guastafierro, S. Viglione, A. Cozzolino, F. Russo, R. Polito, A. Daniele, E. Nigro, M. Ciccarelli, R. Russo

**Affiliations:** 1 Orthopaedic Department, Pineta Grande Hospital Castel Volturno, Caserta, Italy; 2 Dipartimento di Scienze Mediche e Chirurgiche Avanzate, Università Degli Studi Della Campania, “Luigi Vanvitelli”, Naples, Italy; 3 Dipartimento di Scienze e Tecnologie Ambientali Biologiche Farmaceutiche, Università Degli Studi Della Campania “Luigi Vanvitelli”, Via Gaetano Salvatore, 486, 80145 Naples, Italy; 4 CEINGE-Biotecnologie Avanzate Scarl, Naples, Italy

**Keywords:** Athletes, SLAP lesion, Diabetes, Sport, Shoulder physiotherapy

## Abstract

**Purpose:**

The management of isolated SLAP lesions is still debated especially in athletes. Aims of the study were: 1. to analyse our algorithm to treat SLAP lesions starting from the selection of patients for surgery and 2. to correlate the familiarity for diabetes and hypothyroid disorders with post-operative results.

**Methods:**

Seventy-eight patients with isolated SLAP lesion were arthroscopically treated using knotless anchors and microfractures. All patients had a pre-operative and post-operative clinical examination according to Walch–Duplay, Constant, Rowe and Dash scores and interviewed for familiarity to diabetes and hypothyroid disorders.

**Results:**

About 68.8% of patients solved pain with rehabilitation. About 29% of patients returned to the sports activities. About 32% of patients were no responder to physiotherapy and were arthroscopically treated. About 53.9% of patients responded excellent, 34.7% good, 3.8% medium and 7.6% poor results according to Walch–Duplay score. The Constant score increased from 64 to 95, the Rowe score from 48 to 96. The outcomes were significantly worse in patients with familiarity for diabetes.

**Conclusions:**

Microfractures and knotless anchor give long-term good results for the treatment of SLAP lesions in athletes. The familiarity for diabetes is an important risk factor that can lead to decreased outcomes.

## Introduction

The articular part of long head of the biceps and the superior labral complex of the glenoid are important elements that mostly passively participate to the stabilization of the humeral head, in particular in abduction and external rotation. Repetitive activities and efforts in traction are responsible for the most frequent injuries in young athletes [1–3]. The management of isolated SLAP lesions is still very controversial and difficult because several factors have been shown to negatively influence both surgical and non-surgical outcomes: patient’s age, activity level, soft tissue quality, concomitant pathologies such as rotator cuff tear and the surgical technique [1]. In the early 2000s, the isolated or associated SLAP lesions were typically treated arthroscopically in a very high percentage of athletes to quickly recover and go back to sport activities [4–6]. However, the surgical treatment achieved inconstant results in terms of pain, stiffness and return to the same sport level. Published studies at short- and medium-term follow-up (3.1–6.5 years) showed that almost half (49%) of patients with SLAP lesions did not require surgery, suggesting a quite high level of success with non-surgical management [7]. Indeed, a specific rehabilitation programme represents the most valid approach to treat these patients focusing on core and periscapular stabilization, rotator cuff strengthening and neuromuscular control and posterior shoulder stretching [8]. Surgical treatment can include debridement, posterior capsulotomy, anterior capsule plication, superior labral repair, biceps tenotomy, biceps tenodesis or a combination of these procedures.

In addition, recently, several diseases, such as diabetes, thyroid disorders and inflammatory bowel diseases, have been suggested to increase the risk of post-operative stiffness and pain after SLAP repair [9]. In particular, diabetes seems to have additional harmful effects, and emerging evidence suggests that glycation of collagen may be an important factor in stiffness and pain [10]. The purpose of this study was: (1) to define the treatment algorithm in sportsmen (overhead and collision sports) for the treatments of SLAP lesions; (2) to retrospectively analyse the clinical results at long-time follow-up after arthroscopic treatment using absorbable or not absorbable knotless anchors and (3) to evaluate the potential impact of familiarity health disease as the diabetes and hypothyroid on post-operative outcomes.

## Methods

### Patients’ recruitment

From January 2002 to December 2014, 250 athletes practicing collision and overhead sports (135 tennis player, 70 swimmers, one water polo player, three kickboxers, two boxers, five basketball players and 34 bodybuilders) with diagnosis of SLAP lesions were recruited from private database of senior author and three private physiotherapy centres (Table 1). Exclusion criteria were as follows: traumatic or atraumatic anterior or posterior shoulder dislocation and multidirectional instability. All patients had a pre-operative clinical examination according to Walch–Duplay, Constant, Rowe and Dash scores, radiographic study and arthro-MRI. The inclusion criteria were as follows: Young patients aged from 18 to 30 years practicing collision or overhead sports with clinical and imaging doubtful of SLAP lesions, no history of shoulder dislocation, uncertain of minor instability and pain during sport activities. Exclusion criteria were as follows: presence of the following diseases: COPD–stroke–cancers–liver steatosis, BMI > 25, smoker status, anterior or posterior shoulder dislocation, stiffness, arthritis and rotator cuff tear. The average age of all patients was 21 ± 6; 187 males (74.8%) and 63 females (25.2%). The apprehension test, load and shift test, relocation test, O’Brien test 10 and Kim11 test were performed in all 250 patients but the most sensitive test in our experience was the modified dynamic labral shear (DLS) described by Kibler in 2009 [11, 12]. All patients were subjected to a rehabilitation protocol for a 6-month period composed as follows:—to reduce the inflammation, patients received cryotherapy and steroid and NADS drugs;—the passive motion in water at 34/35C°, according to the Lyonnese protocol [13], was used in the 1st month to treat periscapular muscle contractures;—from the 2nd month, a Hawkins protocol was started and focused on the rebalance of rotator cuff with proprioceptive neuromuscular exercises to restore the glenohumeral function in synergy with the scapula position [14].

**Table 1:** General population of athletes

Athletes	Number of athletes	Males/females N	Athletes underwent arthroscopic repair
All sports	250	187/63	78
Tennis	135	102/33	37
Swimming	70	46/24	18
Water polo	1	1/0	0
Kickboxing	3	3/0	2
Boxer	2	2/0	1
Basketball	5	5/0	3
Bodybuilding	34	30/4	17

After the rehabilitation period, 172 patients out of 250 solved the pain and discomfort of the shoulder, while for those still in pain (78 athletes—31.2%), a second arthro-MRI was performed to move to surgical treatment. O'Brien and Kim tests were positive in all patients selected for surgery, 64.1% and 37.3%, respectively. The DLS was positive in 75% of cases, while the 78% of patients were still in pain (Table 2). The mean age of 78 patients suitable for surgery (52 males and 26 females) was 25.4 ± 5 years. The mean follow-up was 124 months (12–156 months). The arthroscopic findings were as follows:—Maffet lesion in 4 cases (5.1%);—SLAP II with an anterior prevalence in 65 cases (83.3%);—posterior prevalence in 6 cases (7.6%) and—SLAP lesion type I in 3 cases (3.8%) [15]. In one case of the Maffet lesion, a section of the biceps and an intra-articular tenodesis was carried out according to Rodosky technique [15]. In most of these patients (85%), a single absorbable knotless anchor was used to stabilize the bicipital anchor after an accurate preparation of the footprint of the biceps in association with microfracture. The post-operative immobilization in sling was for 4 weeks for all athletes. Patients started a passive assisted mobilization exercises in water and passive self-assisted exercises in the scapular plane since the 18th post-operative day. The complete range of motion has been reached on average of 40 days, and sport practice was allowed after 6 months. Walch–Duplay, Constant, Rowe and DASH tests were used to evaluate the clinical results.

**Table 2:** Athletes underwent arthroscopic repair

Athletes underwent arthroscopic repair (78)			
	Before rehabilitation programme	After 6 months rehabilitation	At 10-year follow-up after the surgery
M/F	52/26	–	
Age	25.4 ± 5	–	
Maffet lesion	5.1%	–	
SLAP II with an anterior prevalence	83.3%	–	
SLAP II with posterior prevalence	7.6%	–	
SLAP lesion type I	3.8%	–	
Apprehension test—Positive	5.1%	5.1%	0%
Load and shift test	0%		
DLS	92%	75%	20%
Relocation test	0%		
O’Brien test—Positive	85%	64.1%	19%
Kim test—Positive	80%	37.3%	15%
Pain—Positive	100%	78%	21%

*DLS* Dynamic labral shear

Furthermore, all patients have been interviewed to evaluate the familiar predisposition and/or the presence of diabetes (or pre-diabetes) and thyroid disorders.

Familial predisposition was defined as the presence of one and/or more siblings with diabetes and thyroid disease. Pre-diabetes was determined by glucose levels between 110 mg/dL and − 125 mg/dL and by glycated haemoglobin between 6 and 6.49% for pre-diabetes patients, while diabetes by glucose levels above 125 mg/ml and by glycated haemoglobin above 6.5%. Among all 78 patients, nobody had type II diabetes, and only two patients had thyroid diseases. Thirty patients had a diabetes familiarity in terms of presence in their relatives of glucose levels above 125 mg/ml, 21 treated with oral therapy and nine with insulin. In 21, a thyroid disease familiarity was found, 19 with hypothyroidism and two with hyperthyroidism [16, 17].

### Surgical technique

The surgery was carried out in lateral decubitus with peripheral and general anaesthesia. The upper limb has been positioned with traction of 5 kg with an abduction of 45° and posterior shoulder inclination of 15°. The standard posterior arthroscopic was used to the joint exploration. Standard mid-glenoid and anterosuperior portals were used in 70 cases out of 78 (89.7%). In 8 cases (10.3%), a Wilmington portal was added. In case of types II, III or IV SLAP lesions, the glenoid superior aspect of biceps footprint was carefully debrided to expose the subcortical bone surface using a motorized device. In 85% of patients treated, we used an absorbable anchor at 12 o’clock position (Bioknotless DePuy Mitek, Raynham, US). In all cases, two or three microfractures were made on the glenoid edge peeled especially in the posterosuperior aspect of the footprint. In six cases, with a very large anterior to posterior lesion, it was necessary to use two anchors, one anterior and one posterior, to achieve a more stable repair. In all patients, microfractures were performed in the central and posterior area of the biceps footprint. SLAP lesions were repaired using a penetrating grasper or a clever hook (DePuy Mitek, Raynham, US) introduced through the anterosuperior portal, the carrier wire has been recovered from the anteroinferior portal. In this manner, the wire loop came to be found in the upper cannulated portal and passed through the tissue and finally taken with the knotless anchor (DePuy Mitek, Raynham, US) that will be inserted in the glenoid hole. In 48 cases (61.5%), only one anchor has been used in the centre of the superior glenoid biceps footprint. In 1 case (1.2%), the biceps tendon was detached from the superior tubercle, and a tenodesis was performed according to Rodosky technique. In 11 cases (14.10%), a small supraspinatus and partial infraspinatus tears were found and treated with debridement. In 1 case (1.2%), the lesion was repaired because it was more than 50% of tendon attachment. In 2 cases (2.4%), an exploration of the subacromial space was done, and an acromioplasty was performed.

### Statistical analysis

Statistical analysis was performed using Statistical Package for the Social Sciences (SPSS) v. 20.0 (IBM Inc., Armonk, NY, USA). Data are shown as means ± standard deviation (SD) or as number (percentage). Univariate comparisons of dichotomous data were performed with the use of the Chi-square or Fisher exact test, while the *t*-test was used to compare group means with SD. Two-side *p* values were calculated. A *p* value < 0.05 was considered statistically significative.

## Results

**Fig. 1:**
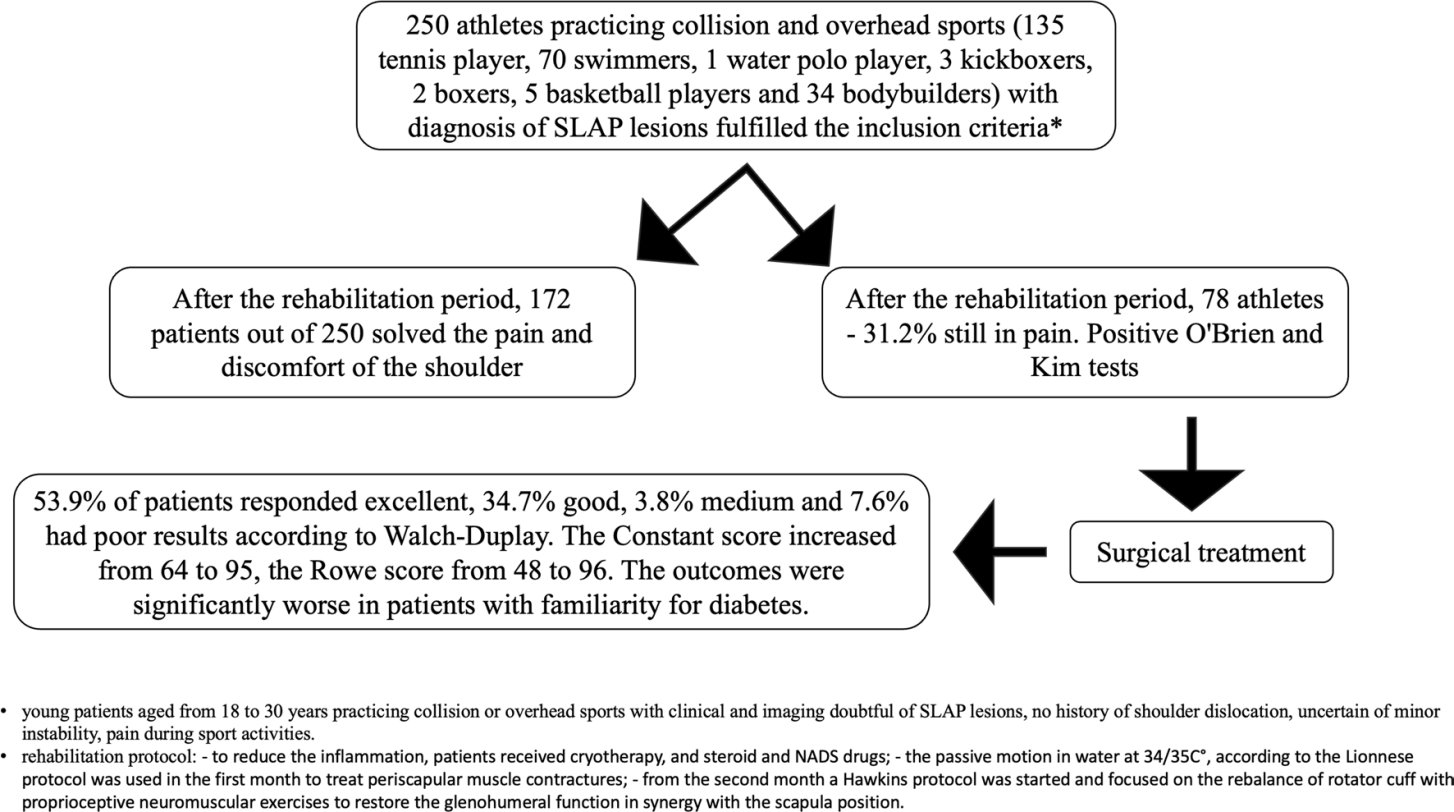
Flow diagram of the cohort study

A consecutive series of 250 patients with isolated SLAP lesions were recruited and followed up: 172 out of 250 patients (68.8%) had an improvement in terms of function and pain after 6 months of rehabilitation programme based on the association of Lyonnese and Hawkins protocols applied in hot water. However, only a small part of patients (29%) treated with specific rehabilitation returned to the same level of sport. These patients were still painless at clinical follow-up at 6 months and 1-year post-rehabilitation (Fig. 1).

Seventy-eight patients (31.2%), despite the physiotherapy, had continuous pain and discomforts and were, therefore, eligible for the arthroscopic treatment (see Table 3). After the surgical treatment, at an average follow-up of 124 months (12–156 months), the Constant score increased from an average value of 64–95 and the Rowe score from an average of 48–96, before and after surgical treatment, respectively. According to the Walch–Duplay score, the clinical results were excellent in 42 cases (72.5%), good in 27 cases (34.6%), medium in 3 cases (3.8%) and poor in 6 (7.6%). The median Dash score was 6.50 (0.80–22.40). All 78 patients returned to sport, 48 (61.5%) at a competitive level while 30 (38.5%) at an amateur level.

**Table 3:** Familiarity in patients treated with arthroscopic repair

	No. of athletes underwent arthroscopic repair/total athletes	% Athletes underwent arthroscopic repair
Familiarity for diabetes	30/78	38.4
Familiarity for thyroid	21/78	26.9

**Fig. 2:**
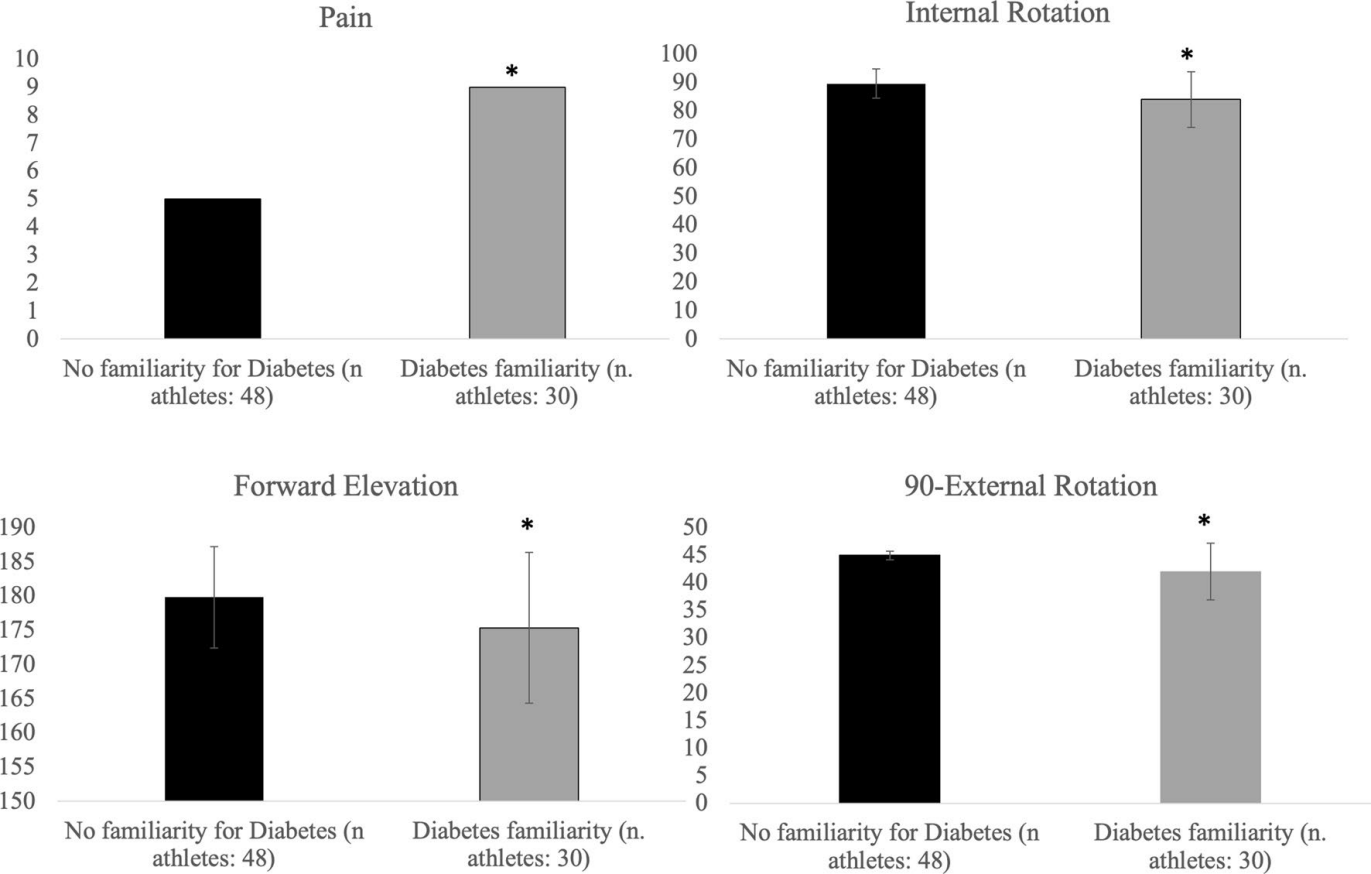
Parameters measured in 78 patients surgically treated with knotless anchor according to the presence of familiarity for diabetes. Data are given as mean ± standard deviation or number (percentage)

We found a strong correlation between diabetes familiarity, post-operative pain and range of motion at short- and long-term follow-up. Pain was present in 9 patients (30%) versus 5 (10.4%) who had no family history; a less forward elevation in 175 ± 8 versus 179 ± 1.4, p: 0.006, intra-rotation in 84 ± 11 versus 90 ± 2.9, p: 0.01 and 90-degree external rotation in 42 ± 5.2 versus 44.9 ± 0.7, p: 0.005 were found (Fig. 2).

Furthermore, females have a stronger correlation between familial diabetes and pain than males. There were no statistical correlations between thyroid disorders or long-term familiarity with SLAP repair in terms of pain or range of motion (Table 4).

**Table 4:** Data are given as mean ± standard deviation or number (percentage)

	No familiarity for hypothyroid diseases (no. of athletes: 57)	Hypothyroid familiarity (no. of athletes: 21)	*p* value
Pain	9 (15.8%)	5 (23.8%)	0.508
Abduction	87.5 ± 7.20	84.00 ± 8.43	0.238
Forward elevation	178.68 ± 4.85	174.00 ± 8.43	0.118
External rotation	87.06 ± 8.47	82.00 ± 12.29	0.238
Internal rotation	88.24 ± 6.45	82.00 ± 12.29	0.148
90-degree external rotation	44.19 ± 3.06	41.00 ± 5.16	0.086

## Discussion

This study describes our approach to the treatment of SLAP lesions, first considering a conservative (rehabilitative) methodology to obtain a functional but painless shoulder and instead considering the surgical approach only for those suffering from post-rehabilitation chronic pain. In surgically treated patients, the diagnosis was confirmed by clinical examinations and arthro-MRI. The best management of isolated SLAP injuries is still difficult to plan, especially in overworked athletes.

Our results demonstrated that rest from sports activities and an adequate rehabilitation programme led to pain reduction in 68% of patients; for those undergoing surgical treatment, our results were positive in over 80% of patients, and no cases of reoperation were reported suggesting that, when the indication for surgery is correct and patient selection appropriate, the accurate technique using knotless anchors and microfractures on the biceps the imprint can persist over time causing the return to the same sporting activities. In accordance with our data, Morgan et al. published a large retrospective review of 102 patients undergoing an arthroscopic repair of SLAP type II lesions with suture anchors [18]. At 1-year follow-up, they reported 97% positive or excellent results. In a study of military patients with type II SLAP lesions, Enad et al. demonstrated a very similar experience with good or excellent results in twenty-four cases out of 27 [19]. This study shows that over 90% of subjects with SLAP lesions were able to return to their full military duty. This would demonstrate that arthroscopic repair of type II SLAP tears with anchors would reliably lead to full return to activity and high levels of post-operative satisfaction, at least at short-term follow-up. Krispi et al. outlined that surgical repair for anterior shoulder instability and a coexisting SLAP lesion yields clinical results as good as those of isolated anterior Bankart repairs (ABR) [20]. Green et al. demonstrated that a combined posterior labral and SLAP repair led to statistically and clinically significant increases in outcome scores and high rates of return to active duty in military patients [21]. Although these published data demonstrate that many technical aspects of arthroscopic repair of type II SLAP tears have been well described, there are controversies on algorithms at the beginning and imaging evaluation, timing of surgery, management of intraoperative lesion according patients age, number and type of anchors to use [22, 23]**.**

For this reason, the clinical decision to perform surgery may not be as clear, and there are little data in the literature, especially on what is the best time for surgery after diagnosis.

The careful selection of patients to undergo surgery was the key to our experience which allowed us to have a number of failures lower than those reported in the literature. Katz et al. [24] in a retrospective study of 40 shoulders in 39 consecutive patients (mean patient age, 43 years) treated for SLAP lesions repair found a poor outcome in 71% of patients with after SLAP repair and completely dissatisfied of conservative treatment and back to surgery. Therefore, once a patient has a poor outcome after SLAP repair, there is a high probability of conservative treatment failure. Although most patients get better results with surgery, 32% of them will continue to have a suboptimal result; in fact, only 29% of them returned to competitive sports [25].

In our study, the excellent results obtained even at a long-term follow-up demonstrate that careful preparation of the biceps impression with microfractures and the use of a knotless anchorage (absorbable or non-absorbable) in the central part of the biceps attachments can improve tissue healing by avoiding local ischaemia [26, 27].

In accordance with us, Blonna et al. [31] reported in a prospective study conducted on 65 consecutive patients scheduled for arthroscopic subacromial decompression, or rotator cuff tear repair with a follow-up was planned at 30, 60, 90 and 180 days after surgery, an overall incidence of post-operative stiffness of 29%.

In this context, it should be considered that there are many metabolic and inflammatory factors that influence the outcome of the intervention. In fact, tissue healing is closely related to risk factors such as family history of diseases such as metabolic disorders that can affect fibrocartilage tissue repair.

SLAP lesion repair can be influenced by several inflammatory mediators such as pro-inflammatory cytokines and other important immunological components. Data from the literature suggest that SLAP lesions might share the same diabetes-related molecular mechanisms such as microcirculatory impairment and non-enzymatic glycosylation processes [28].

In addition, an unfavourable microvascular environment caused by high blood sugar levels can occur around the shoulder joint. Reduced and/or impaired blood flow leads to tissue hypoxia, overproduction of free radicals, which ultimately can lead to a cellular apoptosis process. These damages could lead to joint tissue destruction and potentiation of degenerative changes [29, 30].

Our data show that, on average 6 months after surgery, among athletes with a family history of diabetes, there are poor transient outcomes in terms of pain and stiffness in the immediate post-operative period and with pain persistence and recovery difficulties of full movement. At long-term follow-up in the athletes underwent to surgical repair, the pain was present in 9 with familiarity for diabetes (30%) rather than 5 (10.4%) with no familiarity (p: 0.037), the forward elevation, the internal and external rotation were lower in those with familiarity compared to the other group (p: 0.006). Our clinical results in terms of pain and range of motion are better than those reported in the literature. Our data suggest that SLAP injuries are not only a patho-mechanical lesion of the shoulder, but may be associated with biochemical and immunological factors. On the contrary, we did not find a statistical correlation between family history of hypothyroid disorders in terms of both pain and mobility.

Some limitations of this study should be noted: No control group was present, no histologic study was performed on pathological tissues, it was not possible to perform the MRI in all patients at long-term follow-up in order to see the healing of SLAP repair; furthermore, the potential molecular factors by which diabetes influences the predisposition or development of SLAP lesion have not been investigated. In summary, type II diabetes might be involved in SLAP lesion because it is responsible of the chronic inflammation and also for the recruitment of immune cells in loco of shoulder. Future possible directions concern additional investigations and possible correlations of SLAP outcome with other pathologies and the molecular mechanisms underlying such complex cross-talk.

In conclusion, our data on 250 athletes with isolated SLAP lesion allowed us to understand the most appropriate algorithm to support return to sport or have a painless shoulder or transition to a surgical treatment. In our experience, the 68.8% of cases had good results with a specific rehabilitation programme for 6 months, while the remaining part needed surgery.

We performed a very long-term follow-up in 78 patients surgically treated. Interestingly, the arthroscopic technique using only one central knotless anchor associated with microfracture of the biceps footprint can simplify the SLAP lesions repair reducing time and re-operations and guarantees very good results with a long-term recover in terms of pain and healing. Our data clearly suggest that family history of diabetes is an important factor that can lead to poor outcomes in the post-operative period and long-term follow-up, resulting in residual pain and loss of mobility. Our study contributes to validate the results of the previous studies concerning the identification of some risk factors in the surgical treatment of SLAP. Our data strongly underline the importance of an appropriate pre-operative selection of patients not based only on the lesion. A proper diagnosis of this pathology and the surgical technique must be associated to an accurate clinical investigation of various general aspects of health as an important predictive factor for effective surgical repair.

## Abbreviations

MRI: Magnetic resonance imaging
DLS: Dynamic labral shear
